# Numerical analysis of fiber reinforced composite material for structural component application

**DOI:** 10.1016/j.heliyon.2024.e37698

**Published:** 2024-09-12

**Authors:** Chala Amsalu, Debela Negasa, Amanu Merga

**Affiliations:** a Department of Mechanical Engineering, School of Mechanical and Industrial Engineering, Hachalu Hundessa Campus, Ambo University, P.O. Box 19, Ambo, Ethiopia; b Xinjiang Technical Institute of Physics & Chemistry, Chinese Academy of Sciences, Add:40-1 Beijing Road, Urumqi, Xinjiang, 830011, China; c Faculty of Materials Engineering, Department of Materials Technologies, Silesian University of Technology, ul.Krasińskiego 8, 40-019 Katowice, Poland

## Abstract

Nowadays, convectional metallic material replaced by composite materials, because composite materials have superior than metallic materials properties such as light weights, higher strength-to-weight ratio, high tensile strength, Low cost, greater design flexibility, better fatigue resistance, renewability, and biodegradability. These properties of composite material are the most basic & common attractive features that make them useful for industrial applications. The main objective of this work is to contribute for a better understanding of the static behavior of structure made from fiber reinforced composite materials, specifically for the case of plate structures. The plate model is created using SOLIDWORKS 2017 and then imported into ANSYS R18.1. The study specifically examines three stacking sequences of the composite plate (angle ply, cross ply, and multidirectional ply) to analysis stress and deformation resulted from the loads. The static analysis of a Carbon/Epoxy with honeycomb plate composite reveals that the equivalent stress and deformation are lower in the cross-ply stacking sequence compared to the angle ply and multidirectional ply for the same load carrying capacity. This suggests that the composite plate with a cross ply configuration is more suitable for manufacturing composite structures due to its improved performance.

## 1 Introduction

Composite materials have been used for industrial products for nearly forty years, and they have developed significantly at duration of that period. Especially [1,2],. Furthermore, continuously developed new material systems are giving us infinite possibilities for structural design. Fiber reinforced composite materials are increasingly being used for joining composites to different composites or to metal. Fiber-reinforced composites are commonly differentiating by a high degree of specific strength and specific stiffness parameters (i.e., weight to strength ratios) and are commonly used in low-weight component applications [3]. All practical reinforced plastics composites are likely to contain defects of various kinds arising from the processes of manufacture. The micro structural in homogeneity and anisotropy of fiber composites are together responsible for the fact that the fracture of these materials is rarely a simple process. Although the complex combination of micro-failure events that leads to deterioration of load-bearing ability, can often give rise to surprisingly high levels of toughness, the same complexity makes it difficult or impossible to use procedures based on fracture mechanics for design purposes. The fracturing of a composite therefore involves not only the breaking of the Load-bearing fibers and the weak matrix, but a complex combination of crack deviations along these weak interfaces [4,5]. The high strength and damage resistance of the composites are very important for several practical applications. Many kinds of mathematical models of deformation, damage, and failure of fiber reinforced composites have been designed in order to predict the strength and other characteristics of these composites [5]. The micro mechanisms of damage in fiber reinforced composites (FRC) can be described as follows [6]. When a fiber reinforced composite with a ductile matrix is subjected to longitudinal tensile loading, the fibers bear most of the load and fail first. As the weakest fibers fail, the remaining intact fibers experience increased loading, potentially leading to the failure of neighboring fibers. Cracks in the fibers can cause stress concentration in the matrix, which may result in matrix cracking. However, if the interface between the fiber and matrix is weak, the crack can propagate along the interface. In ceramic and other brittle matrix composites, cracks initially form in the matrix. However, if there are intact fibers behind the crack and they connect the crack faces, a crack bridging mechanism occurs. This mechanism allows the load to be shared by the bridging fibers and the crack tip, resulting in a reduction of the stress intensity factor at the crack tip [7]. When there are more bridging fibers present, the stress intensity factor at the crack tip decreases, resulting in increased resistance to crack growth. As the crack extends and is bridged by intact fibers, the fibers may debond and pull out, which enhances the fracture toughness of the material. This phenomenon contributes to the overall strength and durability of fiber reinforced composites.

**Fig. 1:**
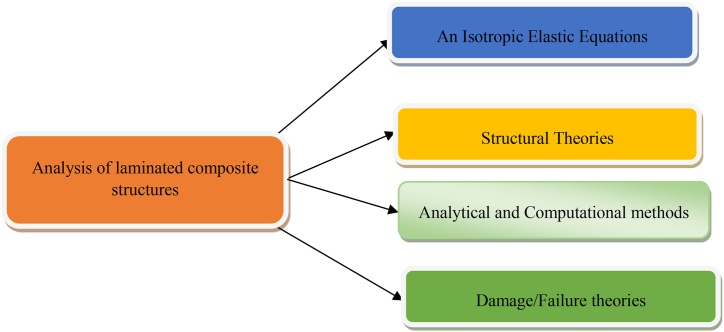
Basic Blocks in analysis of composite materials [8].

Furthermore, analysis of structural made of laminates composite materials involves knowledge anisotropic elasticity, structure theory, of laminates, computational methods to determine solution of governing equation and failure theory to predict the mode of failure and to determine failure loads as shown in Fig. 1 [8]. Finite element analysis of laminated composites and the effect of stacking sequences on laminated composite plates are extensively studied topics in structural engineering. However, achieving the optimal stacking sequence for composite laminate plates in structural engineering applications has not been accomplished yet and requires further research. Currently, researchers are actively working to propose the optimal stacking sequence for laminated composite plates. The main objective of this research is to investigate the impact of stacking sequence on the static behavior of plates and propose the optimal stacking sequence through numerical analysis using the Finite Element method.

## 2 Literature review

### 2.1 Numerical micromechanical analysis methods

In a series of works, the composite deformation and crack growth under transverse loading was simulated using micromechanical finite element models. Firstly, as studied on [9] utilized computer-aided design (CAD) and finite element analysis (FEA) to simulate the behavior of a composite material under single fiber pull-out conditions. They investigated the impact of material and geometric variations on the stresses experienced by the fiber and matrix. The study also explored the effects of different stiffness ratios, fiber cross-sections, and the number of modeling elements on the axial stress and interface shear stress in the fiber and matrix. Likewise [10], developed a multi-scale 3D finite element (FE) model was developed by researchers to study fracture in fiber-reinforced composites (FRCs). The model focused on a notched specimen made of SiC fibers and a Ti matrix, subjected to three-point bending. The simulation considered three damage mechanisms: plastic deformation of the matrix, brittle failure of fibers, and frictional sliding on the interface. In the study, the researchers simulated the fracture of fibers by incorporating randomly placed interface elements along the fibers. These interface elements utilized the cohesive crack model to replicate the fracture process. The interaction between the fiber and matrix was modeled using the elastic contact model, assuming that the interface strength is negligible, and the interaction is governed by friction. The simulation results were then compared to experimental data, showing a favorable agreement between the two. In a similar manner, researchers in Ref. [11] conducted simulations to study the cracking of the matrix in fiber-reinforced composites (FRCs). They utilized digital image analysis to determine the real microstructures of carbon fiber reinforced polymers (CFRPs) and incorporated them into finite element (FE) models using the embedded cell approach. By considering both mechanical and statistical criteria, they obtained probability density functions for stress, strain components, and dilatational energy density in the loaded composites. Other author [12,13] study and compare the buckling load resisting capacity of the dispersed stacking sequences with other plate that have quasi-isotropic stacking sequence (0°, ±45°, and 90°) fiber angle and identical ply thicknesses. The results showed that the dispersed stacking sequences performed better than the plates with regular stacking sequences.

### 2.2 Fiber bundle model analysis methods

Several fiber bundle models (FBM) have been developed to analyze composite materials, considering the matrix, interfaces, nonlinear behavior of fibers, and micromechanics of failure. Firstly, as studied on [14], the researcher developed one model which is the continuous damage fiber bundle model (CDFBM), which incorporates multiple fiber failures. Researchers have used this model to study the scaling behavior of composites and found that the multiple failures of brittle fibers can result in ductile behavior of the composite. Likewise, according to Ref. [15] extended Fiber Bundle Model (FBM) to include the plasticity of fibers when failed fibers still carry a fraction of their load. By incorporating the plastic fiber bundle model, they demonstrate that the failure behavior of the material depends on whether failed fibers continue to bear load, and the macroscopic response of the composite can exhibit plastic behavior if the fibers are plastic and the loads are redistributed according to the GLS schema.

### 2.3 Fracture mechanics analysis model methods

As stated by Ref. [16], the researchers investigated the process of damage initiation and growth in fiber glass ceramic matrix composites when subjected to flexural loading. They developed an axisymmetric damage model that considered an annular crack surrounding a fiber and calculated the energy release rate based on the volume fraction of fibers. This model was then used to analyze the failure modes of glass matrices reinforced by coated SiC fibers, as demonstrated by researcher. In a similar manner, the partial-debonding theory proposed by Ref. [17] was utilized to analyze crack bridging in fiber-reinforced composites. This theory, based on the shear lag model and primarily used for fiber pullout tests, assumes that the fiber/matrix interface consists of a deboned region with linear stress variation along the fiber length and a fully bonded, elastically deforming region. By treating each fiber and its surrounding matrix as a single pullout test, he determined the stresses in the fiber and at the interface, and their model was compared to experimental analysis using Raman spectroscopy to assess the stress distribution in the composite.

### 2.4 Shear lag based model analysis methods

Firstly, as studied [18], the authors of the study used a 3D finite element micromechanical analysis to investigate the deformation and stress transfer in fiber-reinforced composites (FRCs). They utilized the results obtained from finite element modeling to extract the relevant Green's function for a larger-scale model of stochastic fiber damage distribution, focusing on factors such as stress distribution around broken fibers and the average axial stress concentration factor on fibers near the break. n the 3D finite element (FE) model, the researchers used the same hexagonal geometry and microstructural parameters as the shear lag model. They simplified the model to a 30° wedge by considering its symmetry. By comparing the stress distribution, fiber stress concentration factor, and other parameters, they found that the shear lag model accurately represents systems with high fiber/matrix stiffness ratios and high fiber volume fractions, but not those with low fiber volume fractions. Likewise, the author [19] applied the shear lag model to a system with multiple fibers. They investigated the stress distribution around broken fibers in 2D composites with an infinite array of fibers. By extending the elastic model to three dimensions and incorporating the elastic-plastic matrix, they determined the average Stress Concentration Factor in a fiber after neighboring fibers failed. In the context of composite materials, the shear lag model was developed by researchers [20] to investigate how stress is redistributed from a broken fiber to its neighboring fibers. They determined the Stress Concentration Factor (SCF) for the scenario where a single fiber breaks in a 2D unidirectional composite. Their findings showed that the impact of a fiber break on nearby fibers is less severe than previously estimated, depending on factors such as the distance between fibers and the number of adjacent broken fibers.

Based on the review, it can be concluded that the primary approaches utilized to analyze the strength and damage of fiber reinforced composites include the shear lag model, fiber bundle model, and micromechanical unit cell models. When examining the strength, damage, and fracture of these composites, several challenges must be addressed. These challenges encompass accurately representing the transfer and redistribution of loads between fibers and the matrix, considering the interaction between multiple fiber cracks, matrix and interface cracks, and modeling the bonding mechanisms at the interface and their impact on composite behavior.

The transfer of loads from failed fibers to the matrix is commonly modeled using the shear lag model and its variations, direct micromechanical analysis, or phenomenological load redistribution laws. Additionally, micromechanical finite element simulations are frequently employed in conjunction with other methods to supplement, verify, or test research findings [18,21].

## 3 Materials and methods

In this paper fiber-reinforced carbon epoxy resin composite with honeycomb plate material is designed and analyzed using ANSYS R18.1 Workbench Software to meet the final goal of the study that is; taking different laminate stacking sequences plate, comparing the stress in the composite material of each plate and decide which stacking sequence is good for manufacturing of plate materials for different type of purposes.

### 3.1 Materials

**Fig. 2:**
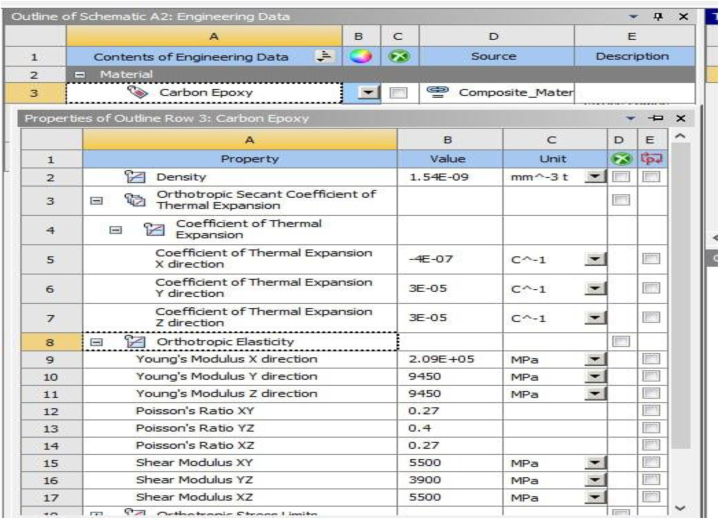
Properties of carbon/epoxy fiber material.

In this present work, three different orientations (angle ply, cross ply and multi directional ply) of structured plate from carbon fiber reinforced epoxy matrix composite material were modeled. Since it has excellent mechanical properties, and light weight nature, Carbo/epoxy material has been selected for this study, and the mechanical properties of carbon/epoxy are illustrated in Fig. 2. The finite element analysis was performed on ANSYS 18.1 workbench. The total deformation, equivalent stress (Von-Mises), and Shear stress for three different cases of fiber-reinforced carbon epoxy resin composite is compared.

### 3.2 Making model of composite plate using ANSYS R18.1 workbench

**Fig. 3:**
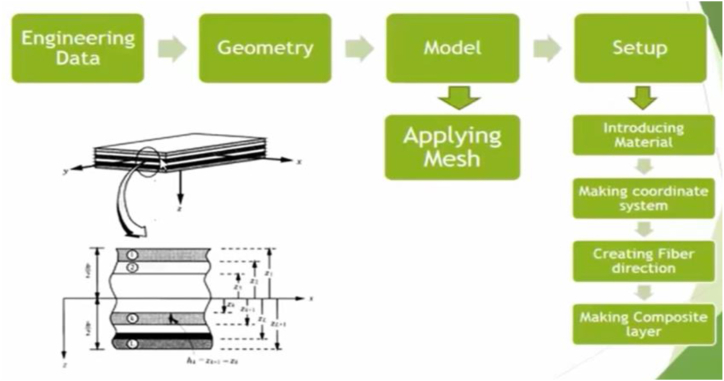
General process of making model (ACP).

The software package used for numerical analysis for this study ANSYS 18.1. The General process of making model (ACP) is shown in Fig. 3.

### 3.3 Modeling of plate

**Fig. 4:**
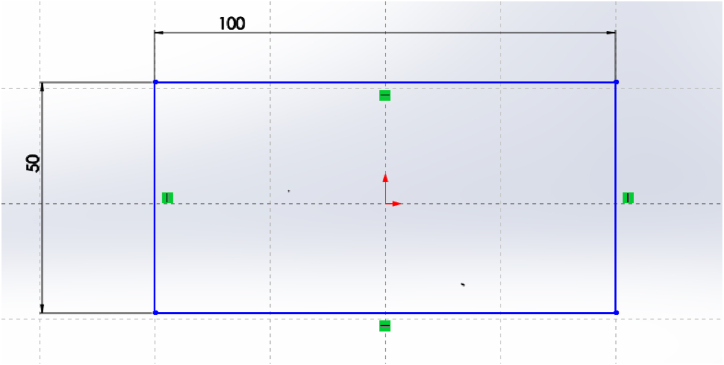
Two-dimensional model of plate.

**Fig. 5:**
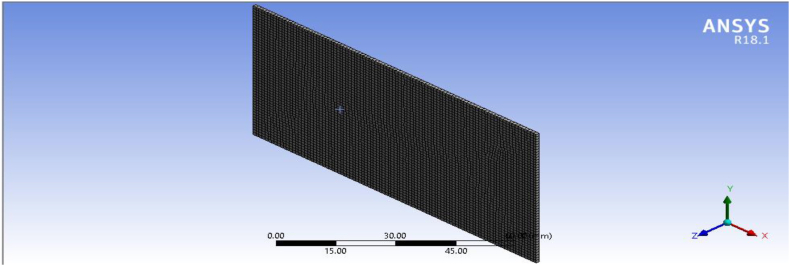
Meshed model.

**Fig. 6:**
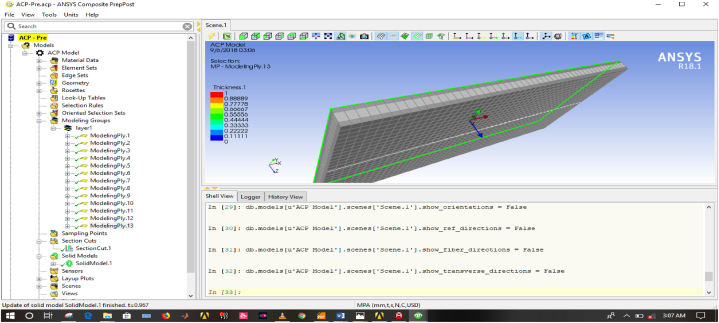
Final model of composite plate.

A 2D sketching of the plate (Fig. 4) was created with the help of SOLIDWORK 2017 solid modeling software and analysis is done by using ANSYS R18.1 workbench to determine stress and deflection induced on the plate. The mesh model of structured plate and final model of composite ACP-Prepost is presented in Fig. 5, Fig. 6 respectively.

#### 3.3.1 Apply mesh controls/preview mesh

Meshing is one of the most important aspects of finite element analysis (FEA) and it is Meshing is the process of discretizing complex geometries into smaller elements. This is due to the accuracy of the FEA results predominantly depending on the mesh size and element quality. The larger the density of meshing (smaller element size and larger number of nodes), the greater is the accuracy of the results. There is different type of meshing method in ANSYS workbench includes: Automatic, Tetrahedrons/Triangles, Hex Dominant, Sweep, and Multitoned. In this work, the tetrahedral meshing approach is employed for all models of structured plate geometry. This is due to Tetrahedrons meshing produce high quality meshing for boundary representation most of solids model imported from CAD systems [22]. Additionally, in this research, Number of nodes and elements for structured plate were 494742, and 320066 respectively.

#### 3.3.2 Generating ply in composite in ACP-prepost

In ACP- Prepost, the orientation of ply, stacking sequence and material properties of each layer was defined and generated. The final model of composite plate is shown on Fig. 6.

#### 3.3.3 Apply loads and supports

**Fig. 7:**
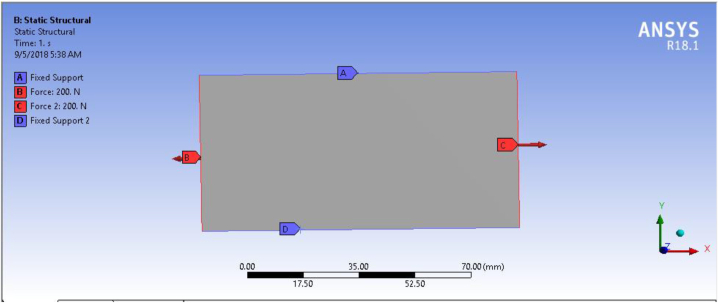
Boundary condition and applied load.

The boundary conditions are collection of different forces, pressure, velocity, supports, and constraints that are required for a complete analysis of any structures. In this article, force and fixed supports are boundary conditions to determine stress in composite plates. The applied force and boundary conditions used in article was shown on Fig. 7.

#### 3.3.4 Generating solution

**Fig. 8:**
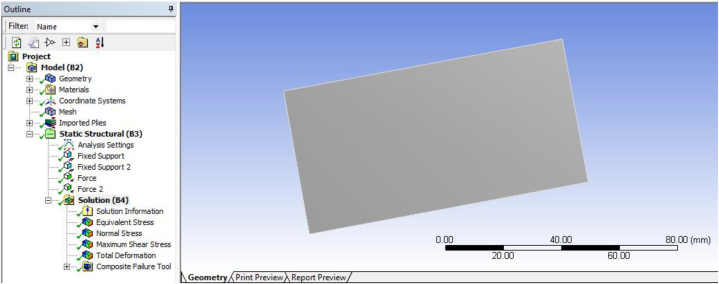
Generating solution of laminated carbon/epoxy with honeycomb plate.

By applying forces and boundary conditions on laminated composite plate of different stacking sequence, total deformation, directional deformation (X, Y, and Z axis), and stress parameters (von misses, and maximum shear, etc …) are solved easily by FEM approach by using ANSYS R18.1 workbench software. In this research the total deformation, normal stress, shear stress and equivalent (von Mises) stress are analyzed as the basic variables and its result shown on Fig. 8.

## 4 Results and discussion

The results of fiber-reinforced carbon epoxy resin composite material from the numerical analysis in ANSYS 18.1 workbench were presented in this work. In the numerical analysis, different laminate stacking sequences is used to perform the analyses, although the geometry and the applied load are the same for all laminate designations. However, this study classified laminate designations into three cases as follow: -

Case 1 Angle ply: [30/-30/30/-30/30/-30/honeycomb/-30/30/-30/30/-30/30]s
Case 2 Cross ply: [0/90/0/90/0/90/honeycomb/0/90/0/90/0/90]s
Case 3 Multidirectional: [0/90/0/90/45/-45/honeycomb/-45/45/0/90/0/90]s

### 4.1 Equivalent (von-mises) stress (MPa)

**Fig. 9:**
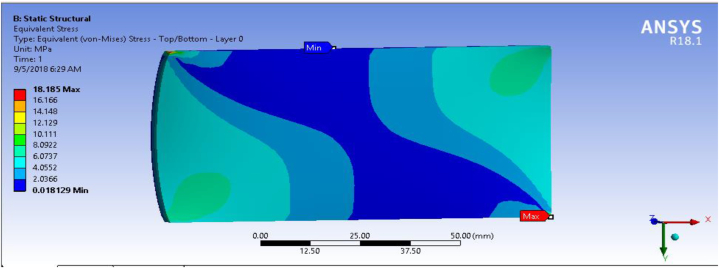
Von-Mises Stress for Angle ply.

**Fig. 10:**
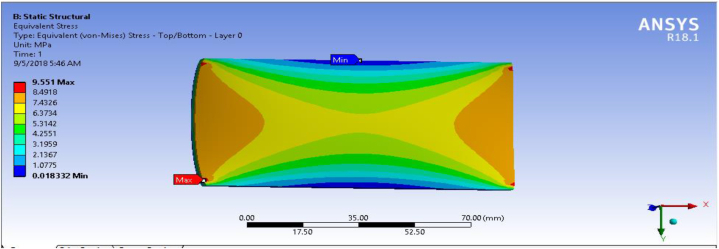
Von-Mises Stress Cross ply.

**Fig. 11:**
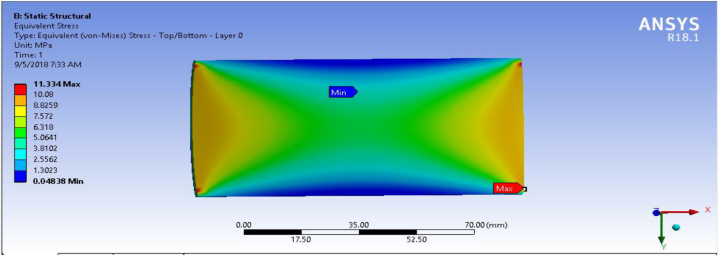
Von mises stress for multidirectional ply.

In this section, fiber-reinforced carbon epoxy resin composite structured plate was modeled in SOLIDWORK necessary results were performed in ANSYS 18.1workbench. Equivalent stress (Von-Mises) for the fiber-reinforced carbon epoxy resin composite with honeycomb structured plate in the direction of angle ply, cross ply and multidirectional were presented and shown in Fig. 9, Fig. 10, Fig. 11 respectively.

### 4.2 Shear stress (MPa)

**Fig. 12:**
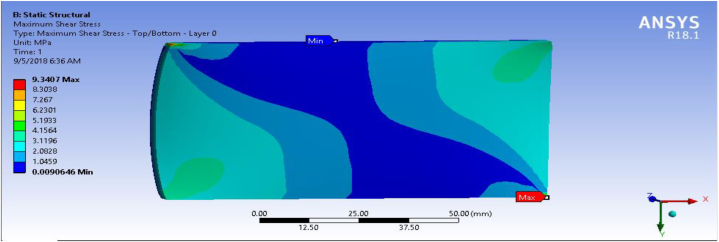
Max shear stress for Angle ply.

**Fig. 13:**
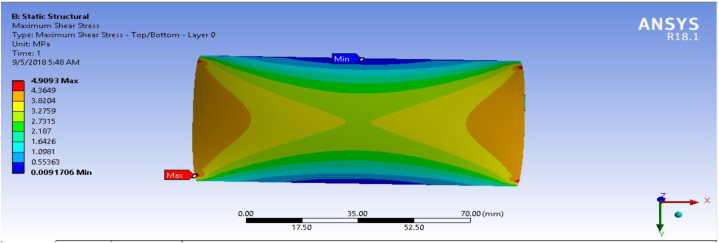
Max shear stress for Cross ply.

**Fig. 14:**
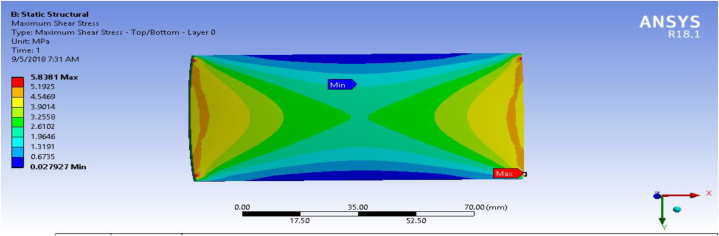
Max shear stress for multidirectional ply.

In this section, shear stress for the fiber-reinforced carbon epoxy resin composite with honeycomb structured plate in the directions of angle ply, cross ply and multidirectional were presented and shown in Fig. 12, Fig. 13, Fig. 14 respectively.

### 4.3 Deformation

**Fig. 15:**
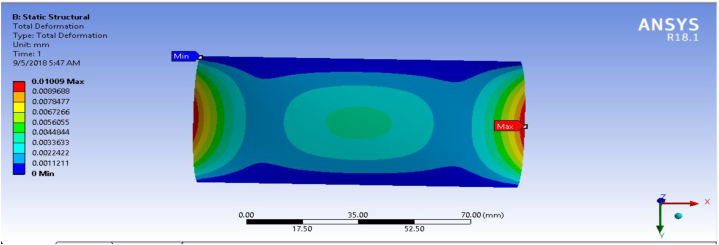
Deformation for Angle ply.

**Fig. 16:**
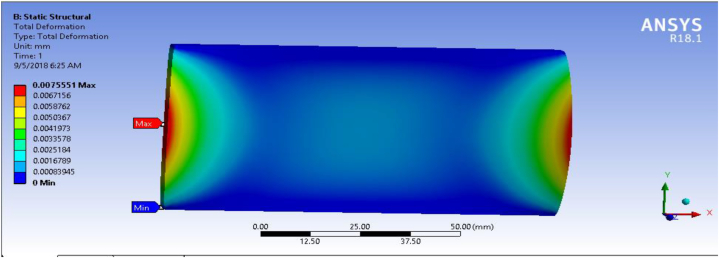
Deformation for cross ply.

**Fig. 17:**
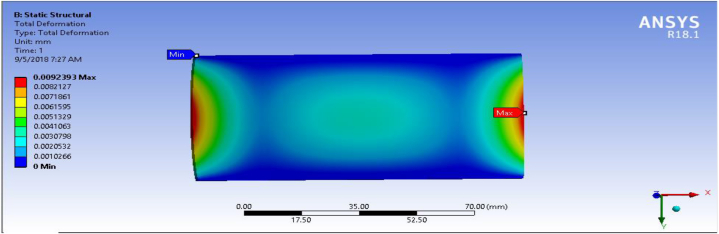
Deformation for multidirectional ply.

In any mechanical system under the applied load, there is always deformation. This deformation should be as small as possible in engineering design. As shown in Fig. 16 the total deformation of fiber-reinforced carbon epoxy resin composite with honeycomb structured plate in the directions of cross ply is 0.0075551 mm. This indicates of fiber-reinforced carbon epoxy resin composite with honeycomb structured plate in cross ply designation has lower deformation in the applied load than angle ply and multidirectional designations were presented and shown Fig. 15, Fig. 17 respectively.

Table 1 shows the comparison of fiber-reinforced carbon epoxy resin composite with three different designations for structured plate. The values obtained from ANSYS 18.1 workbench were through uniform mesh sizes. With relevance center fine, span angel center fine, and smoothing with high the results of equivalent stress and total deformation of fiber-reinforced carbon epoxy resin composite structured plate in angle ply, cross ply and multidirectional designation is 18.285 MPa, 9.551 MPa, 11.334 MPa and 0.011009 mm, 0.007551 mm, 0.0092393 mm respectively.

**Table 1:** Static analysis result summary.

Type of load		Case 1 Angle-ply	Case 2 Cross-ply	Case 3 Multidirectional
Von mises stress (MPa)	Max	18.185	9.551	11.334
	Min	0.018129	0.018332	0.04838
Maximum Shear stress (MPa)	Max	9.3407	4.9093	5.8381
	Min	0.00906	0.00917	0.027927
Deflection (mm)	Max	0.01009	0.007551	0.0092393
	Min	0	0	0

The results of this static structural analysis show that, the value of Von mises stress and Shear stress is observed to be maximum for Case-1 and minimum for Case-2. This implies that plate composite material designated by cross ply is less stressed, light weight and has a better performance. From this static structural analysis, maximum deformation of the composite material plate designated by cross ply plate has the lowest deformation value compare with both angle ply and multi-directional ply.

#### 4.3.1 Load versus von-misses stress

**Fig. 18:**
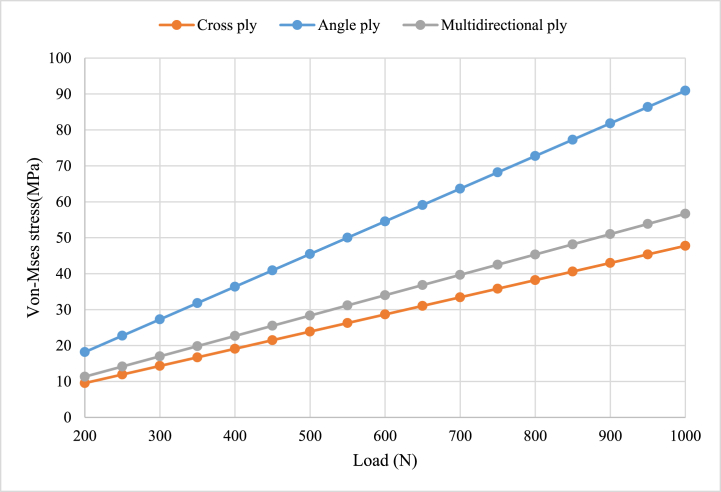
Comparison load versus von-misses stress.

The comparison of load versus von-Mises stress for angle ply, cross ply, and multidirectional orientations of carbon/epoxy structural plates is clearly illustrated in Fig. 18. The x-axis represents the load, while the y-axis represents the stress for angle ply, cross ply, and multidirectional orientations. The observations from the graph indicate varying stress levels for the three orientations under a given loading condition. This suggests that the angle ply and multidirectional ply orientations experience higher levels of stress compared to the cross-ply orientation. This result agrees with a paper published by Ref. [23], Cross-ply laminates exhibit highly stress–strain behavior. In similar manner, the shear strength is also minimum in cross-ply oriented laminated composite plate than angle ply and multi directional oriented carbon-epoxy plate. This result has excellent agreement with the result published by Ref. [24] which stated that, specimens with 0° and 90° axis angles have the minimum in-plane shear strength.

#### 4.3.2 Load versus total deformation

**Fig. 19:**
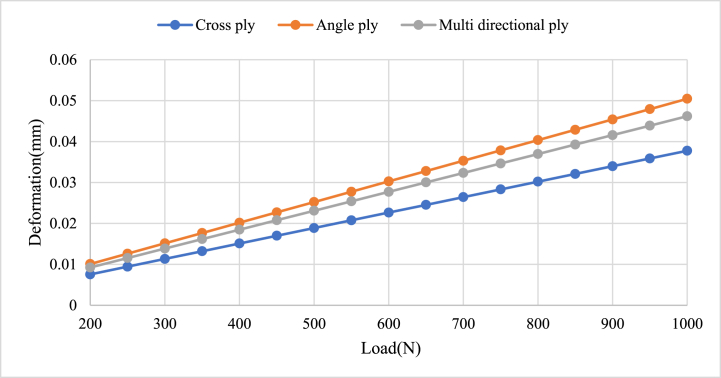
Comparison load versus Deformation.

The comparison of load versus deformation for angle ply, cross ply, and multidirectional orientations of carbon/epoxy structural plates is shown in Fig. 19. The observations from the graph indicate varying levels of deformation for the three orientations under a given loading condition. This suggests that the angle ply and multidirectional ply orientations experience higher deformation compared to the cross-ply orientation. This result agrees with the paper published by Ref. [25], it explores cross-ply laminates exhibit lower deformation compared to angle ply laminates.

## 5 Conclusion

The finite element analysis (FEA) is a powerful computational tool for analyzing complicated Structural bodies. It can reduce prototype parts’ production and the number of physical tests to shorten the development cycle and reduce the development investment, i.e., it saves much time, effort and costs. In conclusion, this study compared the stress and deformation of carbon/epoxy honeycomb plates with three different ply orientations. The results showed that the cross-ply configuration exhibited lower equivalent stress and small deformation as compared with Angle ply and multidirectional ply orientation. In conclusion, this study compared the stress and deformation of carbon/epoxy honeycomb plates with three different ply orientations. The results showed that the cross-ply configuration exhibited lower equivalent stress and small deformation as compared with Angle ply and multidirectional ply orientation. Therefore, from design and static analysis point of view the composite plate designated by cross ply is optimal fiber orientation and better for manufacturing different purposes of composite plate. On the other hand, from basis of the review in the analysis of the strength and damage of fiber reinforced composites, various approaches are used, including the shear lag model, fiber bundle model, and micromechanical unit cell models. However, challenges arise in accurately representing the load transfer and redistribution between fibers and matrix, considering the interaction between multiple fiber cracks, matrix and interface cracks, and modeling the interface bonding mechanisms. The shear lag model, direct micromechanical analysis, and phenomenological load redistribution laws are commonly used to model the load transfer, and micromechanical finite element simulations are often employed to complement other methods. The mechanics of strength and failure in fiber reinforced composites primarily focus on meso-mechanics, which involves the interactions between multiple microstructural elements and microcracks/cracks. Finally, Composite plates with a cross-ply configuration have potential applications in various industries such as aerospace, automotive, marine, and wind energy.

## 6 Future work

➢ Develop advanced manufacturing techniques for efficient and cost-effective production
➢ Study long-term durability and fatigue behavior under various loading conditions
➢ Assess performance of cross-ply plates in different environmental factors
➢ Utilize advanced characterization techniques for better understanding of microstructural behavior and failure

## Data availability

The Von-mise stress, maximum shear stress and deformation data used to support the findings of this study are included within this article.

## CRediT authorship contribution statement

**Chala Amsalu:** Writing – original draft, Validation, Methodology, Investigation, Formal analysis, Data curation, Conceptualization. **Debela Negasa:** Writing – review & editing, Validation, Supervision, Software, Formal analysis. **Amanu Merga:** Writing – review & editing, Validation, Software, Investigation.

## Declaration of competing interest

The authors declare that they have no known competing financial interests or personal relationships that could have appeared to influence the work reported in this paper.
